# A Novel Sample Preparation Method for GC-MS Analysis of Volatile Organic Compounds in Whole Blood for Veterinary Use

**DOI:** 10.3390/ijms26104667

**Published:** 2025-05-13

**Authors:** Kyung-Geun Ahn, Ryuho Choi, Soonchul Gwak, Inyoung Choi, Giup Jang, Jin-Wook Kim, Geon A Kim

**Affiliations:** 1 Department of Biomedical Laboratory Science, University of Health Science, Eulji University, Uijeongbu 11759, Republic of Korea; akg@metadxlab.com; 2 Center for Veterinary Bioinformatics Research and Development, MetaDx Inc., Seoul 04799, Republic of Korea; ryuho@metadxlab.com (R.C.); gsc@metadxlab.com (S.G.); inyoungvet@hanmail.net (I.C.); cto@metadxlab.com (G.J.)

**Keywords:** veterinary cancer, sample preparation, VOCs, mass spectrometry, dog

## Abstract

Volatile organic compounds (VOCs) in biological samples originate both from exogenous and endogenous sources. Recent studies have highlighted their potential as cancer biomarkers, emphasizing the need for accurate detection methods in clinical settings. However, analysis of VOCs in whole blood (WB) samples remains challenging due to the complex matrix effects caused by the protein−VOC binding phenomenon and lack of standardized sample preparation protocols. Therefore, this study suggests a standardized method for advanced VOC analysis in WB samples specifically for veterinary applications. We compared 12 combinations of reagents composed of protein denaturing reagents and salts, particularly urea mixtures, to enhance VOC decoupling from proteins and improve matrix effect uniformity in gas chromatography−mass spectrometry (GC-MS) analysis. Among all combinations, urea with NaCl showed an optimal performance, demonstrating an advancement in the detection sensitivity of up to 151.3% and a significantly reduced matrix effect variation (−35.5% to 25%) compared with the water-only control. This novel approach eliminates complex procedures while maintaining accuracy, making it particularly suitable for veterinary uses. The method’s standardization and improved performance characteristics offer a practical solution for efficient VOC detection in veterinary diagnostics, potentially advancing tumor biomarker research.

## 1. Introduction

Volatile organic compounds (VOCs) are environmental pollutants originating from various industries, vehicle emissions, and other anthropogenic sources [1,2]. Recently, specific VOCs have been identified as potential cancer biomarkers due to their association with altered cancer metabolism, emphasizing the need for accurate detection methods for novel diagnostic solutions. Some studies have suggested multiple VOC biomarkers from breath samples for detecting colorectal cancer [3] and from urine samples for detecting prostate cancer [4,5]. However, in veterinary medicine, these biological samples are difficult to use for quantitative analysis due to the sample quality [6]. Therefore, studies have been conducted to find biomarkers from blood samples for cancer diagnosis in dogs, yet the analysis of VOCs in blood samples remains challenging due to complex matrix effects [7,8].

The matrix effect (ME) refers to the phenomenon in which the sensitivity of an analysis is either increased or decreased due to the matrix contained within the sample [9,10]. To enhance analytical reliability, these MEs are generally corrected or eliminated during the instrumental analysis stage using methods such as standard addition (SA) or matrix matched calibration (MMC) [11,12]. However, as blood exhibits significant variations in concentration and composition among individual samples, the corresponding ME also varies greatly, making it impractical to apply MMC to a representative sample. In other words, to correct for the differing ME in each sample, SA or MMC must be applied to every individual specimen—a process that is extremely cumbersome. Moreover, in cases where the suppression of sensitivity is severe, even correction via SA or MMC may result in analytes being evaluated as undetectable, thereby necessitating the development of approaches to reduce the ME itself during either the pre-treatment or analytical stages.

To minimize ME in blood samples, we selected headspace−gas chromatography−mass spectrometry (HS-GC/MS) as the analytical instrument. This technique analyzes components that have been transferred into the gas phase by heating the sample in a sealed vial [13]. Based on the analytical principle of HS-GC/MS, it was expected that ME would be extremely low during the analysis of VOCs; however, analyses using blood or biological samples still have to confirm the presence of ME [14]. Noting that the target analytes are VOCs and that the sample is blood, we hypothesized that the ME observed during HS-GC/MS analysis of blood is due to VOCs being captured by the abundant proteins in blood through specific interactions.

A study demonstrated that benzene binds stably to hemoglobin (Hb)’s heme pocket, significantly reducing the oxygen binding capacity even at high temperatures (70–80 °C) [15]. In addition, protein aromatic residues and cationic sites have been shown to form strong π−π interactions [16] and cation−π interactions [17]. Also, the influence of ionic strength on aromatic compound solubility suggests that protein−VOC interactions can be modulated by chemical conditions [18]. These studies collectively indicate that VOCs can form specific, stable interactions with blood proteins, which can be disrupted by altering the biochemical conditions, thus providing a theoretical framework for developing improved analytical methods for VOC detection in blood samples.

Generally, when VOC analysis in an aqueous solution is challenging, salts like NaCl are added during the preparation step to decrease the VOC solubility and increase the sensitivity [19,20]. However, to our knowledge, in cases of body fluid samples (serum, plasma, urine, and whole blood), there have been no studies that induce protein denaturation using chemicals to facilitate the release of protein-bound VOCs. Therefore, in this study, we propose a unique method during the sample pretreatment stage that not only enhances sensitivity, but also minimizes the highly variable matrix effects across different matrices, all while being simple, rapid, and highly reproducible.

## 2. Results

### 2.1. Sensitivity Comparison Across Sample Preparation Reagent Combinations

The sensitivity alteration effects among each combination were evaluated by comparing the relative responses to a control containing no additives (Formula (1)). Protein denaturing reagents, urea(U) and SDS(S), were used as additives, while NaCl(NC), K_2_SO_4_(KS), and Na_2_SO_4_(NS) were employed as salts. The specific combinations of protein denaturing reagents (PDR; urea and SDS) and salts that were tested are summarized in Table 1.

Among all of the components, Comb 1(U+NC), 7(NC), and 9(NS) exhibited the most outstanding sensitivity enhancement effect (Table 2). In the case of Comb 7(NC), which showed the most pronounced sensitivity improvement, the enhancement relative to the control group ranged from 196.8% for ethylbenzene to 241.2% for styrene. For Comb 1(U+NC), the sensitivity enhancement for all components ranged from 136.8% to 151.3%, and for Comb 9(NS), it ranged from 126.4% to 169.9%. When comparing Comb 1(U+NC) and 9(NS), benzene, toluene, and styrene in Comb 9(NS) showed sensitivity values of 169.9%, 155.2%, and 154.5%, respectively, which were higher or similar to the values of 143.4%, 147.3%, and 151.3% observed in Comb 1(U+NC). Conversely, in Comb 1(U+NC), ethylbenzene, m-/p-xylene, and o-xylene exhibited sensitivity values of 136.8%, 140.1%, and 147.1%, respectively, which were higher or similar to the 126.4%, 128.7%, and 145.5% observed in Comb 9(NS). Additionally, when evaluating reproducibility through the coefficient of variation(CV) for each combination, Comb 7(NC) showed a distribution ranging from 5.2% to 6.6% for all components, whereas combinations 1(U+NC) and 9(NS) demonstrated superior reproducibility with distributions ranging from 0.8% to 1.1% and 0.5% to 1.0%, respectively.

### 2.2. Sensitivity Alterations According to Sample Volume

The stability of the analytical performance across different sample volumes was assessed by comparing the variability in sensitivity between 0.5 mL and 1.5 mL blood samples, both spiked with 100 ng/mL of the analyte (Formula (2)). This comparison aimed to evaluate the consistency of sensitivity across samples with different matrix levels but identical analyte concentrations (Table 3). Consequently, among the initially selected combinations 1(U+NC), 7(NC), and 9(NS) based on sensitivity comparisons, Comb 9(NS) exhibited high variability exceeding 40% for ethylbenzene (42.8%), m,p-xylene (44.0%), o-xylene (42.9%), and styrene (50.9%). In addition, it showed high standard deviation (SD) values ranging from 16.3 to 21.1 across all components, indicating the lowest reproducibility; hence, Comb 9(NS) was excluded from the list of optimal condition candidates. Conversely, although Comb 2 (U+KS, 10.3–26.5%), 5 (S+KS, 6.3–23.9%), and 10 (U, 4.9–29.2%) exhibited relatively low variability, they were not considered optimal candidates due to their minimal sensitivity enhancement effects compared to the control group.

### 2.3. Comparison of Matrix Effect Uniformity Across Combinations

After confirming the sensitivity advancement and variation, we examined the uniformity of matrix effects according to Comb 1(U+NC) and 7(NC). We aimed to experimentally implement various matrices by using different sample volumes and verified the degree of matrix effects when applying and analyzing each combination across various matrices. Matrix effects were evaluated by measuring the sensitivity changes in samples, calculated by dividing the area values from samples with different blood weights by the area values from samples using water instead of blood (Formula (3)).

**Figure 1:**
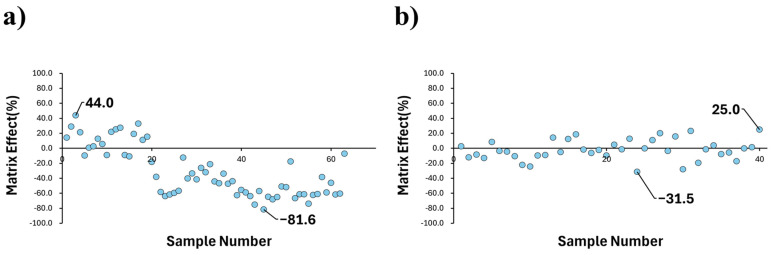
Distribution of fluorobenzene matrix effects with or without the application of Comb 1(U+NC). Matrix effects range from −31.5% to 25%, indicating enhanced uniformity and reduced variability (**b**) compared to the control (**a**).

**Figure 2:**
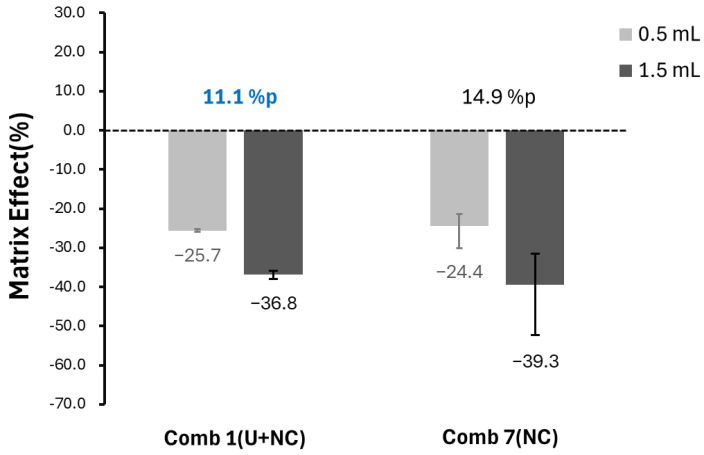
Variance in matrix effects depending on the sample volume according to the combinations.

First, when analyzing 63 blood samples obtained from Naeun Animal Hospital using 1 mL of blood without urea, fluorobenzene’s matrix effects showed a wide distribution ranging from −81.6% to 44% (Figure 1a). Next, the matrix effects were examined for samples of 0.5 mL and 1.5 mL in Comb 1(U+NC) and 7(NC). In Comb 1(U+NC), suppression was observed at 25.7% and 36.8%, respectively, while Comb 7(NC) showed suppression values of 24.4% and 39.3%. Both combinations demonstrated an improved, narrower range of matrix effects compared to the condition without reagent application. Notably, Comb 1(U+NC) exhibited an 11.1% difference between the matrix effects of 0.5 mL and 1.5 mL samples, which is less pronounced than the 14.9% difference observed in Comb 7(NC). Additionally, the variability in repeated analyses across different sample volumes was extremely low for Comb 1(U+NC), experimentally confirming that it allows for a more stable analysis under various matrix conditions (Figure 2).

Subsequently, urea and NaCl from Comb 1—chosen for its minimal matrix effect along with its sensitivity enhancement benefit—were applied to 1 mL samples of various blood matrices. These samples were then analyzed by HS-GC-MS to evaluate the level of matrix effect variability. When analyzing 40 samples, the matrix effects showed a distribution from −31.5% to 25% confirming reduced matrix effects compared to the control (Figure 1b). In short, cases with urea mixtures showed matrix effects more uniformly compared to cases without urea.

## 3. Discussion

Our study demonstrates that protein denaturation combined with salt addition significantly improves the sensitivity and reproducibility of VOC detection in canine blood samples. The wide distribution of matrix effects (−81.6% to 44%) observed in untreated blood samples indicates strong protein−VOC interactions, consistent with previous studies showing stable binding of benzene to the heme pocket [9]. The dramatic reduction in matrix effect variation (−31.5% to 25%) achieved with Comb 1(U+NC) suggests partial disruption of these protein−VOC interactions through the combined action of urea and NaCl. The optimal performance of Comb 1(U+NC) can be attributed to two complementary mechanisms. First, urea’s protein denaturing effect likely disrupts the structural integrity of blood proteins, particularly hemoglobin, releasing trapped VOCs. Second, the addition of NaCl creates a salting-out effect, reducing VOC solubility in the aqueous phase and enhancing their partitioning into the headspace. This dual mechanism not only improves sensitivity, but also provides consistent results across varying sample volumes, a crucial feature for clinical diagnostic applications in veterinary medicine.

While our study demonstrates the significance of protein−VOC interactions in blood analysis, several important aspects remain to be investigated. Although we observed clear effects of protein denaturation on VOC detection, the quantitative relationship between the protein concentration and internal standard response has not been established. Future studies should investigate this correlation to better understand the impact of protein levels on VOC analysis. Additionally, while our focus was on protein interactions, other biomolecules such as DNA and lipids are known to interact with aromatic compounds through various mechanisms. Further research examining the relationships between different biomolecule concentrations and VOC detection would provide a more comprehensive understanding of matrix effects in biological samples.

Nevertheless, the enhanced sensitivity and improved matrix effect uniformity achieved with Comb 1(U+NC) address key challenges in blood VOC analysis. The method’s stability across different sample volumes makes it particularly suitable for veterinary medicine where sample quantities may be limited or varied. Furthermore, the simplified sample preparation procedure, requiring only the addition of urea and NaCl, makes this method both practical and easily implementable in routine laboratory analysis.

## 4. Materials and Methods

### 4.1. Standards and Chemicals

All standard reference materials including benzene, toluene, ethylbenzene, m-/p-xylene, o-xylene, styrene, and fluorobenzene were purchased from Accustandard (New Haven, CT, USA). Stock solutions dissolved in methanol were stored at −20 °C and diluted with methanol as needed. Methanol and HPLC-grade deionized water used for standard dilution were purchased from J.T. Baker (Avantor, Radnor, PA, USA). Urea and SDS (sodium dodecyl sulfate), used to induce protein denaturation, were obtained from JUNSEI (Kosigaya, Saitama, Japan) and Daejung Chemicals (Siheung, Gyeonggi, Republic of Korea), respectively. Among the salts added to facilitate VOC release from blood, NaCl was purchased from Daejung Chemicals (Siheung, Gyeonggi, Republic of Korea), while Na_2_SO_4_ and K_2_SO_4_ were obtained from Junsei (Kosigaya, Saitama, Japan).

### 4.2. Sample Collection

Here, 350 mL canine whole blood packs preserved in a CPDA (citrate−phosphate−dextrose−adenine) bag were purchased from Korea Animal Blood Bank (Goseong, Gangwon, Republic of Korea) and 103 samples were provided by Naeun Animal Hospital located in Euijeongbu, Gyeonggi, Repubilc of Korea, with consent from the owners for research uses. Citric acid or EDTA (ethylene−diamine−tetra acetic acid) were supplemented as anti-coagulants and the samples were preserved at 4 °C until use.

### 4.3. Sample Preparation

PDR along with salts that increase the release intensity of VOCs from samples, as well as water, were added in multiple combinations to a 20 mL headspace (H/S) vial containing the blood sample. For samples treated solely with NaCl, K_2_SO_4_, or Na_2_SO_4_ without PDR, 12 mL of their respective saturated solutions were added. For samples containing urea and SDS, 12 mL of NaCl, K_2_SO_4_, or Na_2_SO_4_ saturated solutions formulated to contain 2.5 M urea and 0.5% SDS, respectively, were added to each sample. The addition of salts induced a salt-out effect, reducing VOC solubility in the aqueous phase and thereby enhancing analytical sensitivity. PDR was formulated by selecting urea and SDS—agents commonly added during protein fragmentation to denature the tertiary structure [21]. These reagents were incorporated to further release VOCs that interact with (or are bound to) proteins in blood, in addition to the free VOCs, through protein denaturation. By transferring VOCs from the aqueous sample phase to the headspace gas in the vial for analysis, the advantages of HS-GC/MS—which eliminates the need for additional processes such as filtration or centrifugation—were fully maximized. This approach greatly simplifies the pretreatment process for practical, routine analyses by merely mixing the blood samples with the reagents.

### 4.4. Gas Chromatography-Mass Spectrometry Analysis

VOCs in canine blood were analyzed using a HS-GC/MS (system equipped with a TriPlus 500 (headspace autosampler) and ISQ 7610 (single quadrupole mass spectrometry) from Thermo Fisher Scientific (Waltham, MA, USA). Sample analysis was performed using 20 mL amber screw cap vials (Thermo Fisher Scientific, Waltham, MA, USA), and the separation of target compounds was achieved using a TG-624SilMS capillary column (30 m, 0.32 mm I.D., 1.8 µm film thickness). The detailed specifications of HS-GC/MS are summarized in Table S1.

### 4.5. Selection of Quantification and Qualification Ions for GC-MS Analysis

Quantification ions were selected from fragment ions showing the highest sensitivity in MS spectra obtained by analyzing standard solutions in positive scan mode. Qualification ions were chosen from ions that showed sufficient sensitivity for compound confirmation and ion ratio verification without interference from other compounds. These ions were analyzed in SIM (selected ion monitoring) mode for quantification, and the detailed mass to charge (*m*/*z*) values are presented in Table 4.

### 4.6. GC-MS Analysis

The prepared samples were heated at 99 °C for 40 min in the instrument’s incubator, after which 1 mL of each headspace gas was injected into the GC-MS. For the separation of VOCs loaded onto the capillary column, the column oven temperature was held at 50 °C for 5 min, then increased to 100 °C at a rate of 10 °C per min and further raised to 120 °C at a rate of 20 °C per min. Subsequently, the temperature was increased from 120 °C to 260 °C at a rate of 30 °C per min and held for 4.33 min, resulting in a total temperature program time of 20 min. Peak integration and quantification of the analyzed data were performed using Trace Finder 5.1 software (Thermo Fisher Scientific, Walthan, MA, USA).

### 4.7. Comparison of Sensitivity and Matrix Effects Among Combinations

Samples purchased from the Korean Animal Blood Bank were analyzed using 0.5 mL for each combination summarized in Table 1 to compare their relative sensitivities. The sensitivity comparison for each combination was performed as a ratio relative to control, which contained no salt or reagent (Formula (1)). Additionally, to indirectly evaluate whether similar sensitivities could be achieved across samples with various matrices, sensitivity changes were measured using blood sample volumes of 0.5 mL and 1.5 mL for each combination in Table 3, with standard solutions added at the same concentration of 100 ng/mL (Formula (2)). Subsequently, after comprehensively reviewing the sensitivity, reproducibility, and sensitivity variations according to sample volume, the two best reagent combinations were selected. The matrix effect was then evaluated by comparing samples without blood to those containing 0.5 mL and 1.5 mL of blood, using fluorobenzene—a commonly used internal standard (Formula (3)). Finally, for the optimal combination, matrix effects on fluorobenzene were assessed using 1 mL of actual samples received from Naeun Animal Hospital.

(1) $$\mathrm{Recovery}\left(\%\right)=\frac{\mathrm{Each}\;\mathrm{Combination}}{\mathrm{Control}}\times 100$$

(2) $$\mathrm{Degree}\;\mathrm{of}\;\mathrm{Sensitivity}\;\mathrm{Change}\left(\%\right)=\frac{\mathrm{Sensitivity}\;\mathrm{in}\;1.5\;\mathrm{mL}-\mathrm{Sensitivity}\;\mathrm{in}\;0.5\;\mathrm{mL}}{\mathrm{Sensitivity}\;\mathrm{in}\;0.5\;\mathrm{mL}}\times 100$$

(3) $$\mathrm{Matrix}\;\mathrm{Effect}\left(\%\right)=\frac{\mathrm{Blood}\;\mathrm{Sample}-\mathrm{Control}\;\mathrm{Sample}}{\mathrm{Control}\;\mathrm{Sample}}\times 100$$

## 5. Conclusions

In this study, we developed a standardized sample preparation method for VOC analysis in WB samples using a combination of urea and NaCl. This optimized method demonstrated significant improvements in both sensitivity and matrix effect uniformity, while maintaining a consistent performance across varying sample volumes. This novel method’s simplified procedure and enhanced analytical performance make it particularly suitable for VOC analysis in veterinary medicine.

## Figures and Tables

**Table 1:** Combination of PDRs and salts used in this study.

Combination(s)	PDR	Salt
Cont.	H_2_O	H_2_O
Comb 1	Urea	NaCl
Comb 2	Urea	K_2_SO_4_
Comb 3	Urea	Na_2_SO_4_
Comb 4	SDS	NaCl
Comb 5	SDS	K_2_SO_4_
Comb 6	SDS	Na_2_SO_4_
Comb 7	H_2_O	NaCl
Comb 8	H_2_O	K_2_SO_4_
Comb 9	H_2_O	Na_2_SO_4_
Comb 10	Urea	H_2_O
Comb 11	SDS	H_2_O

**Table 2:** The sensitivity comparison results across the 12 combinations.

Combination	Benzene		Toluene		Ethylbenzene		m-/p-Xylene		o-Xylene		Styrene	
	Mean ^(1)^	CV ^(2)^	Mean	CV	Mean	CV	Mean	CV	Mean	CV	Mean	CV
Cont.	100.0	3.4	100.0	3.8	100.0	4.1	100.0	4.1	100.0	4.0	100.0	3.7
**Comb 1(U+NC)**	**143.4**	**0.8**	**147.3**	**1.0**	**136.8**	**1.0**	**140.1**	**1.1**	**147.1**	**0.9**	**151.3**	**1.0**
Comb 2(U+KS)	97.3	1.0	102.8	0.9	103.8	1.4	105.3	1.3	104.2	1.3	105.4	1.0
Comb 3(U+NS)	137.5	2.2	140.8	1.3	116.5	0.8	117.1	1.1	130.9	0.8	150.5	0.6
Comb 4(S+NC)	138.3	6.4	115.5	8.3	72.9	8.8	69.9	8.7	79.2	7.8	82.4	6.8
Comb 5(S+KS)	130.2	3.9	123.3	2.3	102.3	2.3	99.7	2.5	101.3	2.8	119.0	2.6
Comb 6(S+NS)	207.1	10.1	148.2	7.3	90.0	10.2	86.8	10.8	101.0	9.1	111.2	10.9
**Comb 7(NC)**	**222.3**	**5.3**	**235.9**	**6.0**	**196.8**	**6.2**	**197.3**	**6.6**	**215.0**	**6.2**	**241.2**	**5.2**
Comb 8(KS)	126.6	2.0	124.5	1.7	122.9	1.9	123.4	2.0	126.4	1.7	119.8	4.6
**Comb 9(NS)**	**169.9**	**0.8**	**155.2**	**1.0**	**126.4**	**0.6**	**128.7**	**0.8**	**145.5**	**1.0**	**154.5**	**0.5**
Comb 10(U)	82.2	2.3	84.5	2.8	85.3	3.0	86.1	2.9	84.0	3.2	89.6	2.9
Comb 11(S)	93.6	9.1	94.9	11.0	85.6	11.0	84.4	10.7	84.5	10.8	53.0	12.8

^(1)^ Mean(%) of triplicate measurements. ^(2)^ Coefficient of variation (%).

**Table 3:** Comparison of sensitivity variations across sample volumes for 12 combinations.

Combination	Mean (%) ± SD ^(1)^					
	Benzene	Toluene	Ethylbenzene	m,p-Xylene	o-Xylene	Styrene
Cont.	12.8 ± 0.7	19.5 ± 0.9	22.8 ± 0.5	23.2 ± 0.5	23.3 ± 0.6	35.0 ± 0.3
**Comb 1(U+NC)**	**16.7 ± 2.2**	**15.6 ± 3.0**	**26.6 ± 2.7**	**28.2 ± 2.6**	**28.5 ± 2.8**	**31.7 ± 1.8**
Comb 2(U+KS)	10.3 ± 0.9	26.5 ± 0.9	14.6 ± 1.0	15.9 ± 0.8	16.6 ± 0.4	24.6 ± 1.6
Comb 3(U+NS)	16.6 ± 5.2	4.3 ± 7.0	43.5 ± 4.7	45.5 ± 4.7	47.7 ± 4.8	44.9 ± 4.7
Comb 4(S+NC)	31.8 ± 6.3	8.4 ± 7.4	39.9 ± 2.8	39.6 ± 2.8	41.3 ± 3.1	39.1 ± 3.9
Comb 5(S+KS)	6.3 ± 1.1	31.2 ± 7.6	13.3 ± 5.0	13.9 ± 5.3	14.4 ± 5.3	23.9 ± 3.8
Comb 6(S+NS)	39.1 ± 3.0	28.1 ± 3.8	48.7 ± 2.3	49.1 ± 2.1	51.2 ± 3.1	47.1 ± 3.0
**Comb 7(NC)**	**19.2 ± 0.8**	**2.7 ± 2.3**	**30.8 ± 1.0**	**31.5 ± 1.0**	**32.6 ± 0.8**	**35.5 ± 1.8**
Comb 8(KS)	18.9 ± 6.7	14.2 ± 4.9	26.2 ± 4.3	27.1 ± 4.4	27.3 ± 3.6	37.8 ± 1.7
Comb 9(NS)	25.4 ± 16.5	17.9 ± 16.3	42.8 ± 20.6	44.0 ± 21.1	42.9 ± 19.1	50.9 ± 18.5
Comb 10(U)	4.9 ± 0.3	34.5 ± 0.7	8.2 ± 0.4	9.4 ± 0.3	10.4 ± 0.2	29.2 ± 0.4
Comb 11(S)	12.8 ± 7.3	54.7 ± 6.0	3.5 ± 1.1	3.9 ± 1.5	4.7 ± 3.1	42.0 ± 5.6

^(1)^ Standard deviation of triplicate measurements.

**Table 4:** The retention time and targeted ions analyzed in this study.

Compound	Structure	Molecular Formula	Molecular Weight	Retention Times	Quantification Ion (*m*/*z*)	Qualification Ion (*m*/*z*)
Benzene	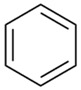	C_6_H_6_	78.11	4.78	78	77
Toluene	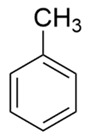	C_5_H_5_CH_3_	92.14	8.08	91	92
Ethylbenzene	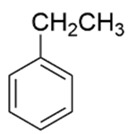	C_6_H_5_CH_2_CH_3_	106.17	10.48	91	106
m-xylene	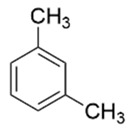	C_6_H_4_(CH_3_)_2_	106.16	10.68	106	105
p-xylene	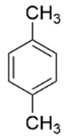	C_6_H_4_(CH_3_)_2_	106.16	10.68	106	105
o-xylene	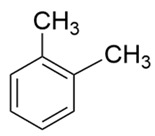	C_6_H_4_(CH_3_)_2_	106.16	11.16	91	106
Styrene	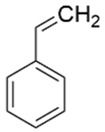	C_6_H_5_CH=CH_2_	104.15	11.24	104	78
Fluorobenzene	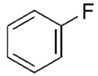	C_6_H_5_F	96.10	5.47	96	70

## Data Availability

Data is contained within the article and Supplementary Materials.

## Supplementary Materials

The following supporting information can be downloaded at: https://www.mdpi.com/article/10.3390/ijms26104667/s1.

## Abbreviations

CPDA	Citrate−phosphate−dextrose−adenine
SDS	Sodium dodecyl sulfate
VOC	Volatile organic compound
NaCl	Sodium chloride
K_2_SO_4_	Potassium sulfate
Na_2_SO_4_	Sodium sulfate
GC-MS	Gas chromatography−mass spectrometry
H/S	Headspace
SIM	Selective ion monitoring
