# Supplementary material for: A Novel Sample Preparation Method for GC-MS Analysis of Volatile Organic Compounds in Whole Blood for Veterinary Use
Source: Int J Mol Sci. 2025 May 13;26(10):4667. doi: 10.3390/ijms26104667 (PMC12111655; doi:10.3390/ijms26104667)
Supplement: Supplementary file 1 [file ijms-26-04667-s001.zip › ijms-3614371-supplementary.pdf]

Table S1. Headspace-GC-MS Analytical Setting Used in this Study

GC	TRACE 1610 (Thermo Fisher Scientific)
MSD	ISQ 7610 (Thermo Fisher Scientific)
Headspace	TriPlus 500(Thermo Fisher Scientific)
Column	TG-624SilMS capillary column (0.32 mm i.d. x 30 m, 0.18 µm film thickness)
Oven(Programming)	Rate 1: from 50℃ (5 min) to 100℃ at 10℃/min<br>Rate 2: from 100℃ to 120℃ at 20℃/min<br>Rate 3: from 120℃ to 260℃(4.33 min) at 30℃/min
Inlet Temp.(℃)	250℃
Septum Purge Flow	2 mL/min
MS Transfer Line Temp.	200℃
Ion Source Temp.	260℃
Carrier gas	Helium, 2 mL/min
Injection mode	Split mode, Split ratio 20
